# A Comprehensive Survey on the Detection, Classification, and Challenges of Neurological Disorders

**DOI:** 10.3390/biology11030469

**Published:** 2022-03-18

**Authors:** Aklima Akter Lima, M. Firoz Mridha, Sujoy Chandra Das, Muhammad Mohsin Kabir, Md. Rashedul Islam, Yutaka Watanobe

**Affiliations:** 1 Department of Computer Science and Engineering, Bangladesh University of Business and Technology, Dhaka 1216, Bangladesh; hossain.limuu@gmail.com (A.A.L.); firoz@bubt.edu.bd (M.F.M.); dsujoy.cse@gmail.com (S.C.D.); mdmkabi@gmail.com (M.M.K.); 2 Department of Computer Science and Engineering, University of Asia Pacific, Dhaka 1216, Bangladesh; 3 Department of Computer Science and Engineering, University of Aizu, Aizu-Wakamatsu 965-8580, Japan; yutaka@u-aizu.ac.jp

**Keywords:** neurological disorders (NDs), computer-aided diagnosis (CAD), machine learning (ML), deep learning (DL), detection and classification, challenges and opportunities

## Abstract

**Simple Summary:**

This study represents a resourceful review article that can deliver resources on neurological diseases and their implemented classification algorithms to reveal the future direction of researchers. Researchers interested in studying neurological diseases and previously implemented techniques in this field can follow this article. Various challenges occur in detecting different stages of the disorders. A limited amount of labeled and unlabeled datasets and other limitations is represented in this article to assist them in finding out the directions. The authors’ purpose for composing this article is to make a straightforward and concrete path for researchers to quickly find the way and the scope in this field for implementing future research on neurological disease detection.

**Abstract:**

Neurological disorders (NDs) are becoming more common, posing a concern to pregnant women, parents, healthy infants, and children. Neurological disorders arise in a wide variety of forms, each with its own set of origins, complications, and results. In recent years, the intricacy of brain functionalities has received a better understanding due to neuroimaging modalities, such as magnetic resonance imaging (MRI), magnetoencephalography (MEG), and positron emission tomography (PET), etc. With high-performance computational tools and various machine learning (ML) and deep learning (DL) methods, these modalities have discovered exciting possibilities for identifying and diagnosing neurological disorders. This study follows a computer-aided diagnosis methodology, leading to an overview of pre-processing and feature extraction techniques. The performance of existing ML and DL approaches for detecting NDs is critically reviewed and compared in this article. A comprehensive portion of this study also shows various modalities and disease-specified datasets that detect and records images, signals, and speeches, etc. Limited related works are also summarized on NDs, as this domain has significantly fewer works focused on disease and detection criteria. Some of the standard evaluation metrics are also presented in this study for better result analysis and comparison. This research has also been outlined in a consistent workflow. At the conclusion, a mandatory discussion section has been included to elaborate on open research challenges and directions for future work in this emerging field.

## 1. Introduction

Healthcare has become a crucial part of the human lifestyle now. Following that, the change and development of healthcare systems have become very dominant in terms of technologies. Identifying diseases has also become very dependent on biomedical technologies, such as ultrasound, X-rays, particle beams, and MRI, etc. With more use of technologies, the excessive growth of biomedical data is a problem for healthcare professionals. Nevertheless, high-computing tools have increased the speed of analyzing biomedical data and reduced work for healthcare professionals. In addition to that, these advancements allowed the researchers to have more audacity to work with more complex clinical patterns. Healthcare further points out disorders of human abnormality, inhibiting or altering the vital functions of several human-body areas. Cardiovascular, genetic, psychiatric, brain, skin, trauma, infectious, tissue, and digestive problems are only a few of the many types of human disorders [1].

**Figure 1:**
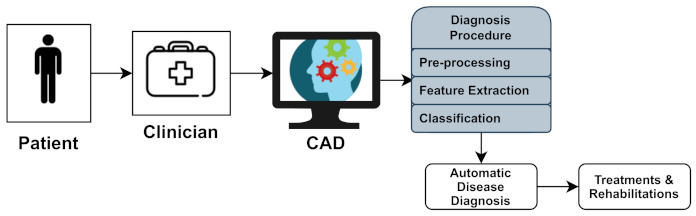
Computer-aided diagnosis (CAD) system architecture.

Neurological diseases (NDs) are a fragment of human disorders that identify complications of the brain. Neurological illnesses, often known as brain, behavioral, or cognitive disorders, affect people’s abilities to walk, speak, learn, and move [2]. As the brain is the control center of the human nerves, affecting the brain can threaten one’s life. Awareness of these diseases has lessened the mortality rate; however, some chronic NDs can cause permanent and partial disability or suffering. The global prevalence of these disorders accounted for 10.2% of the cases. Furthermore, these illnesses have a high causality rate of 16.8 % per year, respectively. These percentages indicate that neurological and neuropsychiatric disorders have higher disability rates than other human disorders [3]. In addition, the diagnosis of neurological illnesses is a developing problem and one of the most complex challenges. For the identification, monitoring, and treatment of neurological diseases, current diagnosis technologies, reviewed in Section 4, produce massive amounts of data. Experts generally perform a manual analysis of big medical data to find and comprehend problems. Recently, an advanced notion of an automated computer-aided diagnosis (CAD) [4] system for experts or neurologists to detect neurological disorders from big medical data has been proposed. The algorithms of significant CAD systems are built using pattern recognition techniques and theories, and consequently, CAD is considered one of the pattern recognition domains [5]. The techniques used by CAD systems are illustrated in Figure 1, and include data pre-processing, feature extraction, and classification. The CAD solutions assist specialists in effectively evaluating big medical data, improving diagnosis accuracy and consistency while reducing analysis time. The CAD system is cost-effective and efficient, and it may be utilized by professionals in the diagnosis and treatment of neurological illnesses as a decision support system. The acquired medical data (e.g., medical image data or medical signal data) were processed during the pre-processing period to remove noise and reduce the complexity and computation time of CAD algorithms. One of the essential elements of the CAD system is the feature extraction section, which extracts disease bio-markers from the source data. The extracted feature vectors are utilized as input in the classifier model for allocating the candidate to one of the available categories (e.g., healthy or normal) based on the output of a classifier in the classification process for CAD systems [6].

However, a few automated computerized categorization approaches for diagnosing neurological illnesses have recently been proposed. They are sufficiently tough to handle data points from various scanners in various applications. Additionally, many developed CAD techniques have been reviewed in a single article. As a result, this study presents a quick overview of some of the essential and recent research on neurological diseases and diagnosing neurological illnesses. Following that, there are some studies, where Nadeem et al. [7] presented an article that aimed to create a significant deep learning concept relevant to brain tumor analysis, reflecting the large variety of deep learning applications. This study looked at brain tumors segmentation, classification, prediction, and evaluation using deep learning. The significant characteristics of this developing subject were reviewed and studied, and a comprehensive taxonomy of the study landscape was based on the existing literature. In addition, Muhammad et al. [8] addressed the fundamental concepts of deep learning-based brain tumor classification (BTC), such as pre-processing, feature extraction, and classification, as well as its accomplishments and deficiencies. This overview outlines the bench-marking datasets that have been used to evaluate BTC. Fundamental problems, such as a lack of public data and end-to-end deep learning techniques, have also been emphasized, and comprehensive suggestions for future research in the BTC field have been made. Shoeibi et al. [9] investigated a wide range of studies centered on automated epilepsy and seizure detection by applying DL approaches and neuroimaging modalities. Several strategies for autonomously diagnosing epileptic seizures utilizing EEG and MRI modalities are outlined. The significant challenges of integrating DL with EEG and MRI modalities to detect automated epileptic seizures accurately were explored. In addition, the most promising DL models were proposed, along with probable future developments. With suitable signposting, Noor et al. [10] showed an overview of different DL designs and pre-processing strategies for detecting anomalies in MRI data, namely a comprehensive review of existing studies based on detection using MRI scans and classification using neural network methods for NDs. In addition, he also provided a comprehensive analyses of accessible datasets, including their origins and extensive data for the subjects (e.g., patients, age, gender, and MRI scan modalities). Yolcu et al. [11] proposed a DL method for automatic facial expression recognition. This paper is the initial step to develop a non-invasive computational system for neurological disease diagnosis, with the primary goal of increasing the quality of service. The proposed framework integrates part-based and holistic information for effective face expression identification. A new framework based on deep learning techniques was suggested (ENDs) by Attallah et al. [12]. The methodology relies on transfer learning and deep feature fusion to recognize ENDs. It utilized raw embryo brain images to develop three deep convolutional neural networks (DCNNs) with distinct architectures. Gautam et al. [3] provided a thorough examination of various deep learning algorithms for diagnosing severe neurological and neuropsychiatric illnesses. This study discovered that EEG- and MRI-based data could be more beneficial for diagnosing epilepsy, stroke, Parkinson’s disease, and Alzheimer’s disease. A summarized information of these related studies are tabulated in Table 1.

**Figure 2:**
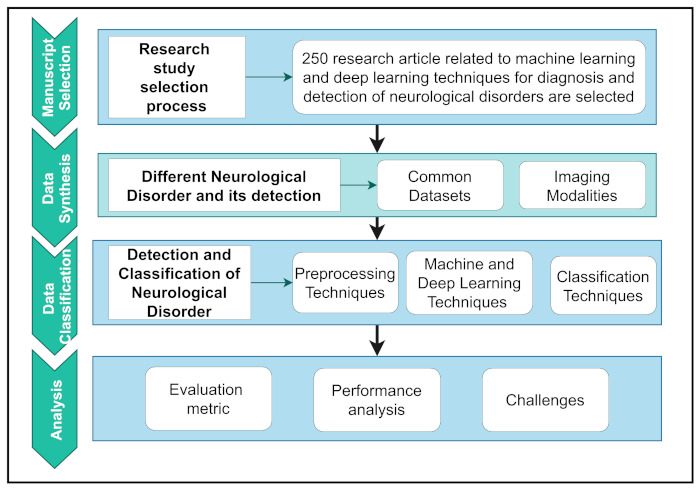
Workflow of neurological diseases detection and diagnosis study.

Here, a thorough study on the prevalence and diagnosis of major human neurological and neuropsychiatric illnesses was conducted using a systematic review of methodologies. Figure 2 depicts the overall workflow of this study.

To organize the workflow, it was first summarized into four separate parts. First, a logical selection methodology is used to extract relevant articles based on research motives. Second, the data synthesis section explored NDs’ datasets and details and the detection of NDs using modalities. Data classification includes a basic introduction and a critical assessment of pre-processing techniques and various ML and DL techniques. Finally, the analysis section shows the evaluation and interpretation of performance analysis and the challenges related to major human neurological and neuropsychiatric disorders. The overall contributions of this study are as follows:

- A concise introduction with the appropriate workflow of the different neurological disease detections of other DL and ML architectures and the pre-processing techniques used in detecting abnormalities from different neuroimaging modalities. It specified the background for a new entrant to the field and was performed as a future reference;
- Thorough interpretation of the existing studies, we reported the purposes and limitations for detecting and classifying neurological diseases. To the best of our knowledge, this is the first attempt to review the ML- and DL-based classification approaches of different neurological disorders from other imaging modalities;
- A comprehensive study on the most popular open-access datasets and their sources, and extensive information on participants in various modalities. We will use open-access datasets to verify and compare the implementation of the proposed technique;
- A robust discussion on recent research issues and future directions to assist entrants in making an impact.

The rest of the paper is organized as follows: Section 2 provides an overview of datasets. A detailed overview of the diseases and their symptoms is reported in Section 3. Section 4 describes the commonly used imaging modalities and their categories. Pre-processing and methods are covered in Section 5 and Section 6, respectively. In Section 7, common categories of machine learning and deep learning techniques are presented. A total of the performance metrics for the analysis of the results of previous studies are presented in Section 8. Finally, an overview of the challenges related to this study is presented in Section 9, and Section 10 concludes the article.

## 2. Dataset

The literature or review on the diagnosis and detection of neurological disorders mainly focuses on techniques, technologies, and results. Therefore, various datasets on neurological disorders are considered vital for a better analysis of these techniques and technologies. However, these datasets also contain specific categories or types. For example, MRI images for detecting neurological disorders and archiving them are vast. Magnetic resonance imaging (MRI) is a non-invasive medical imaging technology for the brain that is utilized to measure and visualize the brain’s anatomical structure, assess brain abnormalities, identify diseased regions, and perform surgical planning and image-guided procedures. MRI pictures are subjected to various image-processing techniques to identify, detect, and classify illnesses and anomalies in the brain. Another popular category is the EEG datasets of brain signals. The electrical activities of brain behaviors were reflected in the electroencephalogram (EEG) data. EEG signals reflect the electrical impulses or disorders of neurons in the human brain. EEG signal investigation is a signal-processing strategy critical for monitoring and diagnosing neurological brain disorders, such as autism spectrum disorder (ASD) and epilepsy. Such actions in the human brain define brain illnesses, such as ASD and epileptic conditions. Currently, brain disorder diagnosis is mainly performed manually by neurologists or competent clinicians by looking at EEG patterns. Parkinson’s disease applications based on speech pattern analysis for developing predictive telediagnosis and telemonitoring models are catching attention. A collection of voice samples was compiled from a set of speaking exercises for people with Parkinson’s disease, comprising sustained vowels, words, and sentences. Two key challenges are learning from a dataset with many speech recordings per participant. First, the accuracy of voice samples of various forms, such as sustained vowels versus words, in diagnosing cases of Parkinson’s disease. Second, the accuracy of the central tendency and dispersion of metrics represents all of a subject’s sample recordings. In addition, the handwriting and facial images of patients with disorders were used to detect diseases. This study has presented various summary tables, Table 2, Table 3, Table 4, Table 5, Table 6 and Table 7 pointing out the number of patients, modality, and available links of datasets of Alzheimer’s disease, Parkinson’s disease, Cerebral palsy, Brain tumor, Epilepsy, respectively.

This study focused on some of the most commonly used datasets in neurological disease detection. These are ADNI, OASIS for Alzheimer’s disease; for Parkinson’s disease, the most frequently utilized is PPMI with a very high number of subjects. Br35H, BraTS (MRI), Temple University EEG corpus dataset has the highest number of subject/patients data suffering from epilepsy. In addition, COBRE [18] dataset of schizophrenia patients is the most common dataset and reliably excellent resource.

## 3. Neurological Diseases

The illnesses of the peripheral and central nervous systems are known as neurological disorders. Muscle weakness, paralysis, convulsions, discomfort, poor coordination, and loss of consciousness are common symptoms. There are more than 600 illnesses that affect the neurological system, including brain tumors, Parkinson’s disease (PD), Alzheimer’s disease (AD), multiple sclerosis (MS), epilepsy, dementia, headache disorders, neuro infections, stroke, or traumatic brain injury. Neuropathological examinations of patients are widely used to identify aberrant or atypical neurological diseases. However, most people have abnormal neurological abnormalities that are not usually linked to a neurological illness [19]. Therefore, a brief review of the conditions and the related parts of the growing severity of the diseases is given in Table 7.

### 3.1. Parkinson’s Disease (PD)

Parkinson’s disease (PD) is one of the most common neurological disorders worldwide, involving one to two individuals per 1000 and with a prevalence rate of 1% in the population over 60 years old [20]. Between 1990 and 2016, the anticipated global population affected by PD more than quadrupled (from 2.5 million to 6.1 million), increasing the number of older persons and age-standardized prevalence rates [21]. PD is a degenerative neurological condition that affects several elements of movement, particularly planning, initiation, and execution [22,23]. Before cognitive and behavioral abnormalities, including dementia, movement-related symptoms such as tremors, rigidity, and initiating problems, can be noted [24]. PD has a significant impact on patient quality of life, social functions, family relationships, and it imposes high financial costs for individuals and societies [25,26,27]. Khojasteh et al. [14] investigated the effectiveness of a deep convolutional neural network (DCNN) in discriminating between PD and healthy voices using spectral data. In addition, the influence of various DCNN architecture designs and characteristics, such as frame size and the number of convolutional layers and feature maps, were examined on raw pathological and healthy voices of differing lengths. Zhang et al. [28] used a PD screening challenge with multiview data, which attempts to employ an MRI data diagnosis to prevent and delay the progression of Parkinson’s disease. They presented a new DL architecture dubbed DNN with broad views to accomplish this goal, based on Wasserstein generative adversarial networks (WGAN), and ResNeXt can influence multiview data simultaneously. Finally, Yuvaraj et al. [29] obtained high-order features using higher-order spectra (HOS) to develop the PD diagnosis index (PDDI), which is a single value that can discriminate between two classes. They also used various classifier techniques to aid clinicians in their diagnosis and help test the efficacy of drugs. The efficiency of supervised classification techniques, such as deep neural networks, in reliably diagnosing people with the condition is investigated in this research(reference [30]). Wodzinski et al. [31] showed how to diagnose Parkinson’s illness using vowels with prolonged phonation, and a ResNet architecture was designed for picture classification. They estimated the audio recording’s spectra and used them as an image input to the ResNet architecture, previously trained with the ImageNet and SVD databases. Tagaris et al. [32] created a novel system that can make predictions and judgments based on a dataset. Their core approach is to use deep learning approaches, which are the state of the art in image analysis and computer vision-based CNN and RNN. The study by Sivaranjini et al. [33] aimed to use a deep learning neural network to categorize the MR images of healthy control and Parkinson’s disease participants. AlexNet, a convolutional neural network design, was utilized to refine Parkinson’s disease diagnosis. The transfer learning network trained and tested the MR images to determine accuracy measures. The research by Shivangi et al. [34] aims to create a deep learning model with two modules: a VGFR spectrogram detector and a voice impairment classifier. These modules use convolutional neural networks (CNN) and artificial neural networks (ANN) to provide a cheaper and more accurate objective diagnosis of PD early.

### 3.2. Dementia

Dementia is linked to the impairment of the elderly all over the world. Dementia affects almost 50 million individuals worldwide, with an estimated 10 million new cases diagnosed each year. Dementia is a syndrome in which cognitive performance, such as thinking, remembering, and reasoning, deteriorates to the point where it conflicts with daily life and tasks. Many dementia patients lose emotional control, and even personality shifts occur. Memory loss, task difficulty, disorientation, language problems, behavioral abnormalities, and lost opportunities for initiative are the most common indications and symptoms of dementia. Dementia symptoms and signs were divided into three stages: early, middle, and late. Due to the steady progression of 226 diseases, the early stage is reasonably vague. It involves losing track of time, amnesia, and becoming lost in familiar surroundings. The middle stage is more evident in events and identities. Additional signs include communication difficulties and an increased need for personal care. With persistent inquiry and roaming, behaviors are altered. The later stage is characterized by atypical symptoms, such as near-total reliance and inactivity due to significant memory problems. Difficulties walking, drastic behavioral changes, failures to recognize time and location, and failures to identify relatives and friends are all detailed symptoms and indicators. The five most common forms of dementia are as follows.

#### 3.2.1. Alzheimer’s Disease

Alzheimer’s disease (AD) is a prevalent type of dementia, and a concern of healthcare in the 21st century. It is a degenerative brain condition characterized by the loss of cognitive function, and is without a proper cure [35]. As a result, much work has been in developing early detection tools, particularly in the pre-symptomatic phases, to reduce or prevent disease progression [36,37]. Advanced neuroimaging technologies, such as magnetic resonance imaging (MRI) and positron emission tomography (PET), have been established and used to detect structural and molecular bio-markers associated with AD [38]. However, integrating large-scale, high-dimensional multimodal neuroimaging data has become difficult because of the rapid advancements in neuroimaging techniques. Lodha et al. [39] created a machine learning model that can reliably forecast a person’s risk of AD based on a set of characteristics that include both cognitive and medical aspects. Ebrahimighahnavieh et al. [40] developed a systematic literature review on DL to detect AD from neuroimaging studies. A review of a new ML method for identifying Alzheimer’s disease is also shown by Liu et al. [41].

#### 3.2.2. Frontotemporal Dementia

Frontotemporal dementia (FTD) is a rare type of dementia that affects behavior and communication and is detected in individuals aged <60. FTD is associated with aberrant levels or types of tau and TDP-43 proteins. The most typical symptoms are extreme personality changes, such as swearing, theft, or worsening personal cleanliness standards. Behavior patterns that are socially improper, impulsive, or recurrent with impaired judgment, lack of empathy, and lack of self-awareness are symptoms of this disease [42].

#### 3.2.3. Lewy Body Dementia (LBD)

Lewy body dementia is a type of dementia characterized by Lewy bodies and aggregates of alpha-synuclein. Following Alzheimer’s disease, it is the second most frequent type of progressive dementia. It grows in the nerve cells in the parts of the brain that control thinking, memory, and movement (motor control). It differs from Alzheimer’s disease, which has less serious memory problems and more significant impairments in visuospatial, attentional, and frontal-executive skills [43]. Only a post-mortem brain autopsy could validate the probable diagnosis of LBD. However, researchers are looking for techniques to detect LBD earlier in life and more accurately.

#### 3.2.4. Vascular Dementia (VD)

Vascular dementia (VD) is a broad term that refers to reasoning, planning, judgment, memory, and other thought processes [44]. This is generally caused by brain damage resulting from reduced blood flow to the brain. It is a chronic condition encompassing a wide range of cognitive dysfunctions produced by brain tissue damage induced by vascular diseases [45]. VD is also a serious concern because of its significant incidence and absence of effective treatments [46]. Though cognitive impairment caused by stroke usually improves with time, vascular dementia caused by SVD is often progressive. Therefore, brain scans such as computerized tomography (CT) or magnetic resonance imaging (MRI) are usually performed on someone thought to be carrying VD to detect any alterations in the brain.

#### 3.2.5. Mixed Dementia

Mixed dementia is a disorder in which the brain shows signs of more than one types of dementias. The most prevalent forms are plaques and tangles associated with Alzheimer’s disease and blood vessel alterations due to vascular dementia. When FTD is combined with motor neuron disease, dementia progresses significantly more quickly with a mobility problem. The typical life expectancy for persons with both illnesses was 2–3 years after identification.

### 3.3. Multiple Sclerosis

“Scar tissue in various places” is the definition of multiple sclerosis. A scar or sclerosis forms whenever the myelin sheath vanishes or is damaged in many locations. These regions are also known as plaques or lesions. The brain stem, cerebellum (which regulates movement), balance, spinal cord, optic nerves, and white matter in specific brain areas, were affected. It is a potentially fatal brain and spinal cord condition (central nervous system). In MS, the immune system attacks the protective sheath (myelin) that surrounds nerve fibers, causing communication issues between the brain and the rest of the body. Four types of MS are generally seen. The first is a clinically isolated syndrome (CIS), with symptoms persisting for at least 24 h. Relapse–remitting MS (RRMS) was the most frequent one. It appears with episodes of new or worsening symptoms, followed by the symptoms being subsided partially or entirely during periods of remission. Thirdly, primary progressive MS (PPMS) cases are characterized by the persistent worsening of symptoms with no early relapses or remissions. Fifteen percent of patients with MS had PPMS. Lastly, the secondary progressive MS (SPMS) initially shows relapses and remissions in patients, regardless of whether the disease proceeds slowly. Shoeibi et al. [47] reviewed DL techniques and the applications of automated MS detection using MRI. Ye et al. [48] also developed a study for the classification of multiple sclerosis lesions on deep learning using diffusion-based spectrum imaging. In addition, an imaging-based machine learning approach to predict conversion from a clinically isolated syndrome to multiple sclerosis was proposed by Zhang et al. [49].

### 3.4. Cerebral Palsy (CP)

Cerebral palsy is a collection of neurological illnesses that begin in infancy or early childhood and impact physical movement and muscle coordination for the rest of one’s life. Damage or abnormalities in the developing brain create CP, which weakens the brain’s capacity to control movements, maintain posture, and balance. Palsy is related to the impairment of motor function, and cerebral refers to the brain. It affects the brain’s motor region’s outer layer (also known as the cerebral cortex), which controls muscular action. Zhang et al. [50] described the application of supervised machine learning algorithms in the classification of the sagittal gait patterns of cerebral palsy in children with spastic diplegia in their study. Bertoncelli et al. [51] identified factors associated with the autism spectrum disorder in adolescents with cerebral palsy using artificial intelligence (AI). The medical diagnosis of cerebral palsy rehabilitation using eye images in ML techniques was proposed by Illavarason et al. [52].

### 3.5. Brain Tumor

A brain tumor is a collection of abnormal cells called neurons that form a mass. There are many distinct types of brain tumors. Certain brain tumors were benign (noncancerous), whereas the others were cancerous (malignant). The indications for a brain tumor vary based mainly on tumor size and location. Many tumors infiltrate the brain tissue and inflict direct injury, while others damage the surrounding brain. Missing borders, noise, and low-contrast factors affected brain tumor segmentation in medical image processing. MRI segmentation utilizing learning algorithms and patterns recognition technologies for analyzing brain data is particularly effective. The technique is a parametric model that considers functions chosen based on the density function [53]. With modern clinical imaging modalities, the early detection of these brain tumors is critical for accessible therapy and healthy living. Particle emission tomography (PET), MRI, and computed tomography (CT) are the most popular modalities used to examine brain tumors [54]. Anil et al. [55] proposed a brain tumor detection method from a brain MRI using deep learning that classified into two classes: with tumor and without tumor. Wu et al. [56] used an artificial intelligence algorithm to diagnose pregnancies complicated by brain tumors using ultrasonic diagnostics. The role of AI in the study of pediatric brain tumor imaging was also investigated in a comprehensive review by Huang et al. [57].

### 3.6. Epilepsy and Seizures

Epilepsy is a neurological condition characterized by recurrent seizures. It is a prevalent long-term mental illness. A seizure is an abrupt shift in behavior caused by a moment of alteration in the brain’s electrical activity. Typically, the brain sends out small electric signals regularly. This produces epileptic seizures, which are electrical bursts in the brain. Epilepsy can be classified into four types: focal, generalized, combination-focal, and undetermined. The kind of seizure a person experiences depends upon the type of epilepsy they experience. When a seizure occurs while an EEG is recorded, the usual pattern of brain activity is disrupted, and unusual bradycardia patterns emerge. When a seizure occurs, EEG is recorded, the regular pattern of brain activity is disrupted, and unusual brain activity can be observed. The electrodes on the brain area where the seizure is occurring can show brain changes during focal seizures. Kaur et al. [58] provided a synopsis of studies on the application of AI systems for real-time pattern detection in EEG for the clinical diagnosis of epileptic seizures. By replicating brain network dynamics, An et al. [59] evaluated artificial intelligence and computational methodologies for the automatic diagnosis and optimal treatment for each epilepsy patient.

With the detailed analysis of NDs and their symptoms, the main focus is detecting NDs with AI. For detection, images of the brain or other parts of the nervous system are required, which is briefly described in the following section.

## 4. Neuroimaging Modalities

Neuroimaging modalities are screening procedures used to diagnose neurological diseases. For clinicians and neurologists, functional neuroimaging is critical information regarding brain function during the development of any condition [60,61,62]. Specialists can learn a lot about persons who may have neurological issues using structural neuroimaging techniques [63]. The most used neuroimaging modalities include magnetic resonance imaging (MRI) [64], electroencephalography (EEG) [65], magnetoencephalography (MEG) [66,67], positron emission tomography (PET) [68,69], single-photon emission computed tomography (SPECT) [70,71,72], functional MRI (fMRI) [73,74,75], computed tomography (CT) [76], and functional near-infrared spectroscopy (fNIRS) [77,78,79,80], etc. In the following subsection, we discuss the 370 most commonly used neuroimaging modalities.

### 4.1. Magnetic Resonance Imaging (MRI)

MRI is the best clinical procedure for diagnosing and analyzing various diseases, including brain tumors and epilepsy [81,82]. Typically, a system controlled by hardware or computers aids in automating a procedure to produce precise and timely results. It is a painless and secure test that employs a magnetic field and radio waves to create high-resolution two-dimensional or three-dimensional images of the brain stem. The brain, spinal cord, and vascular anatomy have been described. Some advantages include witnessing anatomy in all three planes: axial, sagittal, and coronal. MRI outperforms CT in detecting circulating blood and cryptic vasculature abnormalities. It can also detect demyelinating disease and does not have the beam-hardening artifacts of the CT images. The posterior fossa was more prominent. As a result, MRI allows for a better visualization of the posterior fossa than CT. Ionizing radiation was not used during the imaging process.

### 4.2. Electroencephalography (EEG)

Electrical activity on the skull is recorded using EEG, and brain neurons play an influential part in stimulation. EEG is the most widely used method for studying the brain’s functional anatomy throughout neurological disorders and collecting brain activity. This is prevalent because of its superior temporal resolution, safety, and affordability [83,84]. These non-Gaussian and non-stationary signals are used to determine the type of brain disease by covering the brain’s electrical activity. Implementing either inbuilt amplifiers or external amplifiers has the primary goal of reducing the influence of ambient noise. The readings can distinguish normal and pathological brain processes. Experienced Neurologists investigate epilepsy by analyzing continuously recorded EEG signals. One of the problems with EEG is that it requires gels or saline liquids to reduce the skin–electrode resistance. In addition, it requires a significant amount of human effort and time over days, weeks, or even months.

### 4.3. Magnetoencephalography (MEG)

MEG scanning, or magnetoencephalography, is a brain imaging technology that detects and analyzes small magnetic fields created in the brain [85,86,87]. The scanning produces a magnetic source image (MSI), which was used to identify the beginning of the seizures. MEG also monitors current flow in the brain to estimate the magnetic areas. Electric fields go through the skull more frequently than magnetic fields, and they have a better spatial resolution than EEG. The brain’s magnetic field was measured and evaluated using a neuroimaging approach. It works on the outside of the head and is now routinely used in clinical treatment. MEG has grown increasingly important, particularly for individuals suffering from epilepsy and brain malignancies. It could help discover brain regions with normal functions in epilepsy, tumors, or other mass lesions. MEG captures motions with an extremely high temporal and spatial resolution as well. As a result, scanners must be placed near the brain’s surface to detect the cerebral activity that produces small magnetic fields.

### 4.4. Positron Emission Tomography (PET)

Positron emission tomography (PET) is a functional imaging modality that visualizes with radioactive chemicals called radio tracers [88,89,90]. It is a high-tech imaging technology that examines brain activity in real-time and accomplishes the non-invasive monitoring of cerebral blood flow, metabolism, and receptor binding. A PET-CT scan combined 3D-generated images for a more precise diagnosis. Initially, PET was utilized only in research due to the comparatively high costs and complexity of the associated equipment, including cyclotrons, PET scanners, and radio-chemistry laboratories. Owing to technological advancements and the ubiquity of PET scanners, PET scanning has been increasingly used in clinical neurology in recent decades to enhance our knowledge of illness etiology and facilitate diagnosis.

### 4.5. Functional Magnetic Resonance Imaging (fMRI)

Functional magnetic resonance imaging, or functional MRI, defines brain activity by distinguishing the variations in blood flow. The concept that cerebral blood flow and neuronal activity are connected is the foundation of this method. When a part of the brain is used, blood flow to that part of the brain increases. Because fMRI has a high spatial resolution, it is useful for detecting active brain regions [91]. The fMRI method has a low time resolution of one to two [92]. It also has a low head-movement resolution, which might cause distortions. Scans from fMRI are based on the same atomic physics principles as MRI scans. On the other hand, MRI scans depict anatomical structures, whereas fMRI scans measure metabolic function. As a result, the MRI scan results resemble three-dimensional depictions of anatomic structures. It is used to track the progression of brain cancers, assess how well the brain functions after a stroke or Alzheimer’s diagnosis, and detect where seizures originated in the brain.

### 4.6. Functional Near-Infrared Spectroscopy (fNIRS)

Similar to fMRI, functional near-infrared spectroscopy (fNIRS) is a non-invasive brain imaging technology that monitors variations in blood oxygenation [93]. The approach detects variations in the absorption of light emitted by sources onto the surface of the head and is monitored by the detectors. Any brain surgery requires extra oxygen. Capillary red blood cells provide this extra oxygen to the neurons and increase blood flow in the active brain regions.

### 4.7. Computed Tomography (CT)

One of the most often-utilized diagnostics in neurology is computed tomography (CT) [76]. In the 1970s, it revolutionized neurology by allowing the high-resolution viewing of cerebral structures. MRI has primarily replaced CT in the examination of several neurological conditions. However, it still plays a role in the crucial evaluation of stroke and head trauma patients. It assesses head trauma, severe headaches, dizziness, and other symptoms of an aneurysm, hemorrhage, stroke, and brain tumors using specialized X-ray equipment.

### 4.8. Single-Photon Emission Computed Tomography (SPECT)

A single-photon emission computed tomography (SPECT) scan is an imaging examination. This illustrates how blood flows through the tissues and organs [70,94,95]. Seizures, strokes, stress fractures, infections, and spinal malignancies may all be diagnosed using the test. This scanning technique combines computed tomography (CT) and a radioactive tracer to produce a nuclear imaging scan. Experts can see how blood travels to tissues and organs using the tracers. It is primarily used to examine blood flow through the brain’s arteries and veins. It may detect diminished blood flow in wounded regions. It has been demonstrated to be more responsive to brain damage than both MRI or CT scanning in tests. This test differs from a PET scan. The tracer remains in the bloodstream of humans instead of being absorbed by surrounding tissues, limiting the images to areas where blood flows. SPECT scans are cheaper and more readily available than higher resolution PET scans.

The images or brain signals extracted from these modalities contain so much noise that they must be removed to classify better. The following section shows a standard number of pre-processing techniques.

## 5. Pre-Processing Techniques for Neurological Disease Detection

Pre-processing improves the quality of experimental data and prepares it for statistically significant analysis [10,96]. Neuroimaging modalities from various origins consist of noise, including mobility, average signal intensity, and spatial distortions. To provide proper analysis, this troubling amount of noise and other artifacts must be eliminated from the dataset. Brain extraction [97], histogram normalization [98,99], and co-registration [100] are among these processes.

### 5.1. Normalization (NM)

Normalization [101] is similar to image registration. It coordinates and warps the present image data into a size and form similar to a generic anatomic template. Normalization interprets the brain MRI on a standard shape and size by comparing different brain MRI scans. It converts data from a discrete topic space to a reference space that includes a template and source pictures. Most deep learning methods normalize the image intensity with zero mean and unit variance. Normalization can be accomplished using various ways: advanced normalization tools (ANTs) [102,103], standardization [104], intensity normalization [105,106], spatial normalization [107,108,109], Z-score normalization [110,111], statistical parametric mapping (SPM) [112,113], and numerical normalization, were used.

#### 5.1.1. Histogram Normalization

Histogram normalization is a typical technique for enhancing fine detail in an image [114]. The summation of the representation intensity histogram values, including the grayscale values, was determined for each column in the cumulative histogram. After then, it is scaled to a final value of 1.0. A histogram matching method was presented to address changes in scanner sensitivity owing to variances in scanner performance [98,99]. Using this strategy, differences in white matter (WM) intensities may be reduced from 7.5 to 2.5%.

(1) $$g^{\prime }\left(x,y,z\right)=\frac{HIR-LIR}{S_{\max}-S_{\min}}\left(g\left(x,y,z\right)-S_{\min}\right)+LIR$$

If the target histogram of the input image g(x,y,z) starts at $S_{m}in$ and extends up to $S_{m}ax$ grayscale levels, it can be scaled up between the lower and the upper boundaries. This results in voxels in the new normalized image $g^{\prime }\left(x,y,z\right)$ lying between a minimum level (LIR) and maximum level (HIR). The lower and higher boundaries of the reference image before scaling up are represented by the variables $m_{1}$ and $m_{2}$.

#### 5.1.2. Spatial Normalization

This entails deforming each patient’s brain image to accommodate a standardized (template) brain image. Eliminating global differences in the size and orientation of each ’normalized’ brain and ensuring that the same anatomical regions in each image occupy the same voxels, results in lower statistical variance and higher power. It manipulates the MRI scans into a stereotyped space so that the location of one MRI scan resembles that of other MRI scans from the same patient [107,109,115,116,117,118].

#### 5.1.3. Intensity Normalization

Intensity normalization is a crucial step in the study of brain magnetic resonance images (MRIs) [105,106]. Several scanners or parameters might be utilized to scan other people or the same subject at different times during MR image acquisition, resulting in considerable intensity fluctuations. It is used to decrease the intensity variance generated by scanning various subjects or the same subject using different scanners or parameters [119,120].

#### 5.1.4. Z-Score Normalization

A data normalization approach defines the divergence of sample data from the distributed methods to prevent outlier concerns [121]. Imagine utilizing Z-score normalization to transform the data into a more straightforward format. In this situation, our brains have no trouble comprehending it [110,111].

#### 5.1.5. Numerical Normalization (NNM)

This refers to using a mathematical function to transform numerical numbers into a new range. It helps to compare distinct empirical values on multiple scales, allowing their relationship to shine clearly [101].

### 5.2. Filtering

Filtering is a method for altering or improving the images. It is a neighborhood operation generated by running an algorithm on the values of pixels in the vicinity of the matching input pixel. Filtering in image processing is often used to attenuate either the image’s high frequencies, smoothing it out, or the image’s lower frequencies, boosting or detecting the edges. There are a variety of filtering techniques, including spatial filtering (SF) [122], temporal filtering (TF) [123], Wiener filtering (WF) [124,125], and high-pass filtering (HPF).

#### 5.2.1. Spatial Filtering (SF)

Spatial filtering is a technique for modifying the qualities of an optical image by deleting the specific spatial frequencies that comprise an object [126]. This is a pixel-by-pixel picture-enhancement approach. The value of the filtered current pixel is determined by both itself and nearby pixels [122,127].

#### 5.2.2. Temporal Filtering (TF)

A temporal filter determines the spatial placement of the pixel values. It recognizes the collocated reference pixel at least one prior frame. It eliminates frequencies of interest from the raw signal, resulting in a significant increase in signal-to-noise ratio (SNR) [123,128,129].

#### 5.2.3. Wiener Filtering (WF)

Rician noise is a common signal-dependent disturbance found in MRI images. Wiener filtering is a recommended technique for reducing the Rician noise. However, it is also an MSE-optimal stationary linear filter for images split with frequency components and blur. Therefore, the Wiener filter must be calculated based on the hypothesis that the signal and noise processes are both second-order stationary.

#### 5.2.4. High-Pass-Filtering (HPF)

A high-pass filter (HPF) is an electric filter that allows signals above a specific cutoff frequency to pass while attenuating sounds below that frequency. For instance, low-frequency fluctuations in fMRI data might be observed, distinguished by physiological and physical noise. If not addressed, these signal drifts could have a massive effect on statistical data processing. In this case, the high-pass filter feature was used to cut frequencies below a known threshold underneath the lowest frequency.

### 5.3. Stripping

A preliminary stage of MRI analysis is skull stripping, or brain extraction [130,131]. Skull removal is an important pre-processing stage that removes non-brain tissues from brain MRI scans [132]. Several clinical applications and data analyses also require stripping or brain extraction. Additionally, a practical way for improving data analysis speed and experimental accuracy was determined by automated skull stripping. The FMRIB Software Library’s (FSL) brain extraction program and the optimization of the multiplicative intrinsic component are frequently used for skull stripping [133].

### 5.4. Scaling

Scaling is measuring and assigning numbers to items based on predetermined standards. Scaling, in other terms, is the process of situating measured items on a continuum, a continuous sequence of numbers to which they are assigned. Image resizing, image registration, resolution improvement, correction, and other difficulties in MRI scans require modifications.

#### 5.4.1. Image Resizing (IRE)

Image resizing is needed to increase or decrease the total amount of pixels in an image. In contrast, it remaps when it compensates for lens distortion or rotating. The pixel information in an image is modified when it is scaled [134,135].

#### 5.4.2. Image Registration (IR)

IR is a technique for aligning several images in medical image analysis to validate the spatial correlation of anatomy across distinct photos. Linear and non-linear are two types of registration algorithms. Linear registration (Lrg) is global and uses either a six-parametric rigid transformation or a 12-parametric affine transformation (rotation, translation, scaling, and shearing on the x, y, and z axes). Non-linear registration, on the other hand, tends to elevate the extent of the elasticity and local deformation of the model [136,137,138].

#### 5.4.3. Distortion Correction (DC)

The fMRI sequences are sensitive to magnetic inhomogeneity (T2*) effects because they detect gradient echoes. It affects the anterior temporal and frontal lobes, causing dropouts of signals around the foundation of the skull and spatial distortions. Field mapping, unwarping, and correction of phantom-based distortion are some of the ways to reduce these distortions [139].

#### 5.4.4. Contrast Enhancement (CE)

The CE method was employed to contain histogram clustering to rectify the distribution. CLAHE, a CE approach, was used in reference [140].

#### 5.4.5. Bias Correction and Bias Regularization (BC, BR)

A low-frequency biased signal mostly contaminates the MRI images. Notably, a variety of bias correction methods can be applied, as they are produced by older MRI equipment [116,117,133].

### 5.5. Correction

Slice timing correction and motion correction are crucial pre-processing techniques for correcting image slice-dependent delays and subject motion, respectively [141].

#### 5.5.1. Motion Correction (MC)

Head motion is the most common cause of error in fMRI research and is addressed during valuable data acquisition. Unwanted variance in voxels was also introduced by trivial head motions, thereby lowering data quality. Motion correction decreases the impact of movements on the picture data by orienting the data to a reference time volume, as explained in references [142,143,144]. The MCFLIRT module of the FSL library was used to correct motion [115,145].

#### 5.5.2. Slice Timing Correction (STC)

In most fMRI investigations, not every slice in a volume is acquired simultaneously. This means that the signal captured from one portion may be off by up to several compared with the signal recorded from the other [142]. As a result, the temporal discrepancies between the slices must be considered. For slice timing correction, there are two primary solutions to this. The most typical method is data shifting, which involves moving the recorded points to consider their proper offset from the moment of incites.

### 5.6. Smoothing

Smoothing is a technique of eliminating noise in a picture and producing a less pixelated image as a result [33].

#### Spatial Smoothing (SS)

The average of the signals from adjacent voxels is spatial smoothing. It improves the SNR while lowering the spatial resolution, obscuring the image, and smudging started areas onto adjacent voxels. The technique can be challenging since nearby voxels’ functions coordinate blood supply. Spatial smoothing attempts to cope with the variability of functional anatomy that has not been addressed by spatial normalization (“warping”), thereby improving SNR. The user must specify the kernel width in mm “full-width half max” when doing spatial smoothing using a spatially stationary Gaussian filter [115,118,142,143,145,146].

After the pre-processing stage of the basic structure of ML or DL classification models, an initial set of raw data analysis consists of various groups of subjects and their dimensionality. Then, the process of reducing these data into more manageable groups and dimensions named feature extraction is described in the following section.

## 6. Feature Extraction Techniques for Neurological Disease Detection

The primary feature extraction aims to obtain further information from the raw signals by transforming extensive data into fewer feature vectors. Feature extraction approaches are used to extract features with several varieties. Several of them are included in the following section.

### 6.1. Discrete Wavelet Transform (DWT)

The DWT disintegrates a signal into many groups [147], and each is a time series of coefficients characterizing the signal’s time development in the appropriate frequency band. It decomposes a signal into a collection of finite-length basis functions called wavelets, enabling specific signal properties to be targeted in time. DWT is called multi-analysis (MRA) and simultaneously preserves both time and frequency information. Different frequency bands provide helpful information for image processing. Sharp edges of the images are found in the highest bars, whereas the global characteristics are distributed in low-frequency bands. Hence only the approximation band is retained, and the remaining band is eliminated [148]. Several researchers have utilized DWT for their feature extraction [149,150,151,152,153].

### 6.2. Discrete Cosine Transform (DCT)

The discrete cosine transform (DCT) facilitates the separation of an image into portions of different relevance in terms of visual quality. It transforms a signal or image from the spatial domain to the frequency domain, similar to the discrete Fourier transform (DFT). It represents the sum of the sinusoids with various frequencies and amplitudes. This transform focuses the majority of the signal power in a tiny portion of the domain, which in the DCT domain is proven to be the upper-left corner of the transformed image [148]. As a result, fewer coefficients were measured by estimating the original signal, resulting in sparse features. Researchers have utilized the DCT in references [154,155,156,157].

### 6.3. Linear Discriminant Analysis (LDA)

LDA is a technique used to reduce dimensionality in supervised learning. The goal is to maximize the distance between every class while minimizing the spread of the class [158]. Consequently, LDA employs both within-class and between-class measures. Each class will have generally distributed the discriminant parameters. Dimensional data are transformed into a line in a given direction for a two-class problem. The projection direction was chosen based on a variety of factors. Fisher’s linear discriminant aims to maximize the ratio of between-class to within-class dispersions. The authors in references [159,160,161,162] utilized LDA in their research.

### 6.4. Principal Component Analysis (PCA)

PCA is a superior statistical technique to extract features and reduce dimensionality. It employs an orthogonal transformation to reduce a large number of associated smaller sets of linear variable values [163]. PCA had the highest volatility characteristic that contained the most information about specific classes. A p-dimensional dataset is represented in a smaller set of n dimensions, with the idea being that each with n lead the eigenvectors of the global covariance matrix [164]. Researchers have used PCA in references [165,166,167,168,169].

### 6.5. Independent Component Analysis (ICA)

ICA is a computational approach to split multivariate signals into additive sub components in signal processing [170]. This is accomplished by assuming that the sub components are statistically independent and possibly non-Gaussian signals. It emphasizes mutually independent components. The spatial ICA or temporal ICA domain is the common imposing independent component for neuroimaging analysis. The cerebral activity is sparse over several voxels, and spatial ICA is more commonly utilized in fMRI studies. Consequently, autonomous components isolate as many coherent networks as feasible. However, the problem arises with the assumption of sparsity as spatial because ICA splits each non contiguous activity cluster into independent components. Although scalp recordings have unique time courses, ERP data are frequently utilized by temporal ICA. The underlying components are temporally independent but can overlap spatial topographies [171]. Researchers have utilized ICA in references [172,173,174,175,176].

### 6.6. Statistical Features

Several statistical measures have been used to extract while extracting neurological features. These are skewness, kurtosis, and peaks. Some of these are as follows.

Skewness: Skewness is a time-domain metric that gauges the symmetry of a signal around its mean. It can have one of three values: positive, negative, or zero. This is defined as follows:

(2) $$\;\mathrm{Skewness}\;=\frac{E\left[{\left(S\left(n\right)-\bar{S}\right)}^{3}\right]}{\sigma^{3}}$$

where *s* and *E* represent the mean, standard deviation, and statistical expectation, respectively. Skewness is negative when the left distribution is more pronounced than the proper distribution and vice versa. It exhibits zero skewness when both are equal.

Kurtosis: Kurtosis determines whether the data are heavy or a light-tailed normal distribution of the EEG signals. Heavy tails and a noticeable peak near the mean were expected when kurtosis was strong, while the low kurtosis has light tails and a flat top near the mean rather than an intense peak, and the high kurtosis has a sharp peak. For a genuine discrete signal *s*(*n*), the kurtosis is defined as:

(3) $$\;\mathrm{Kurt}\;=\frac{m_{4}\left[S\left(n\right)\right]}{m_{2}^{2}\left[S\left(n\right)\right]}$$

where $m_{i}\left[s\left(n\right)\right]$ is the *i*^th^ central moment of *s*(*n*)

(4) $$m_{i}\left[s\left(n\right)\right]=E\left[{\left(S\left(n\right)-\bar{S}\right)}^{i}\right]$$

### 6.7. Hilbert–Huang Transform (HHT)

The Hilbert–Huang transform (HHT) obtains instantaneous frequency data by decomposing a signal into an intrinsic mode and a trend [177]. It is an adaptive technique with a wide range of stoppage criteria used in various applications, including geophysical and biomedical signal processing. Empirical mode decomposition (EMD) and the intrinsic mode function (IMF) are two processes of the HHT (IMF). The self-distinct oscillation formed from the original data is a distinguishing feature of EMD. The signal is in the self-oscillation phase, and the IMF can detect every change. The zero-crossing and the number of local extremes must be the same, or the dissimilarity must be one, to obtain the IMFs. Several researchers employed the HHT extraction process in their work, as seen in references [178,179,180,181,182].

### 6.8. Wavelet Entropies

Wavelet-based entropies determine the information-related features of a signal. For non-stationary signals, entropy was used to determine how the signals are laid out. Norm entropy, sure entropy, threshold entropy, Shannon entropy, and logarithmic entropy are some of the entropies that are employed. The sure entropy is a type of wave that can be calculated using the discrete wavelet transform (DWT) [183]. Threshold Entropy is a method for determining the number of times the signal exceeds the p threshold in a certain period [184]. Because Parseval’s theory corresponds to the signal’s frequency of the Fourier transform, it is a unified theory for estimating its energy despite specifying its time domain [185].

### 6.9. Hybrid PCA-NGIST Method

The hybrid PCA-NGIST feature extraction method incorporates the PCA approach with the GIST descriptor after normalization, using the L2 norm and resulting in a PCA-based normalized GIST feature extraction method. Two studies of Gumaei et al. [186,187] introduced a normalized GIST (NGIST) descriptor as an improved version of the original GIST descriptor. The NGIST can use the L2 norm to overcome the problem of variations for image illumination and shadow, respectively. It is a low-dimensional representation used to summarize image orientations and scales, offering a rough depiction of normalized data without segmentation. PCA is a typical feature extraction and reduction method, and it builds a new compact set of relevant features from the original GIST features. Thereby it can avoid the overfitting problem in the classification stage. The PCA-NGIST approach calculates GIST features from brain pictures. It determines the eigenvectors with the highest eigenvalues, then projects them onto a new feature subspace with the same or fewer dimensions. Some other research work utilized this extraction method recently in references [188,189].

### 6.10. Histogram of Oriented Gradients

The histogram of oriented gradients (HOG) defines all the aspects of an image’s objects. HOG is a feature descriptor algorithm that uses many occurrences in localized sections to identify the tumor region [190,191,192,193].

After applying these various feature extraction techniques, the basic structure of the detection model led to the classification of data points. The primarily used classification algorithms that predict a particular outcome based on a given input from the test data are described in the next section.

## 7. Classification Algorithms for Neurological Disease Detection

Artificial intelligence (AI) has touched every element of human life, and neurology is no exception. The purpose of this study was to instruct medical practitioners on the relevant aspects of artificial intelligence, namely machine learning and deep learning, to review the development of technological advancements equipped with AI, and to explain how machine learning can revolutionize the control of neurological diseases [194]. A concise description of ML and DL algorithms is further given in this section.

### 7.1. Machine Learning Algorithms

**Figure 3:**
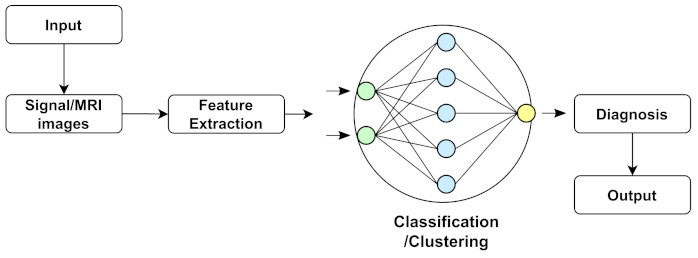
Representation of common machine learning structure.

Machine learning is a branch of artificial intelligence (AI) that allows computers to learn and improve independently without explicitly being programmed. Machine learning is concerned with creating computer programs that can access data and learn independently. Researchers are using machine learning approaches to find statistical patterns in massive datasets to solve a range of problems, including those in neuroscience. In some sectors, recent improvements have resulted in an explosion in the scope and complexity of issues to which machine learning can be applied, with accuracy that rivals or exceeds that of humans. Machine learning (ML) has recently gained popularity for medical disease diagnosis owing to its ease of implementation and high accuracy [195]. As a statistical probability, ML also makes stage predictions of NDs. An ML-based technique was used to determine the actual region to be operated on during ND brain surgery. Some researchers employed machine learning to predict the tremor level of patients with NDs and quantify the cognitive implications of NDs. A common structure of data analysis or classification of ML methods is shown in Figure 3. In addition, some commonly used machine learning algorithms to detect NDs are described below:

#### 7.1.1. Support Vector Machine (SVM)

The SVM uses a non-linear mapping function to map the input data into high-dimensional areas called feature spaces [196]. They determine the appropriate hyperplane for separating the data. SVMs execute linear modeling after projecting the data into another space, whereas traditional linear modeling is performed in the input space. In most cases, functioning as a “black box”, SVMs face problems in interpreting a model’s logic. Therefore, it was a cutting-edge model until its NN architecture outperformed it. In addition, SVM models can adapt effectively to imaging-specific tasks, such as anomaly detection, by utilizing a one-class SVM. One class of SVMs also contributed to medical applications to address the problems with brain tumor detection [197,198]. Some related studies about SVM in the field of neurological diseases are tabulated in Table 8.

#### 7.1.2. Gaussian Mixture Models (GMM)

Gaussian mixture models (GMM) are probabilistic models utilized in supervised and unsupervised learning. According to the model hypothesis, data can be represented as a weighted sum of finite Gaussian component densities. Two parameters characterize each density component: a mean vector and a covariance matrix. Component parameters are calculated using the “expectation maximization” (EM) algorithm, maximizing the component densities’ log-likelihoods. Drawing from the calculated mixture of Gaussian densities was used to achieve this inference. Due to its capacity to represent a vast class of sample distributions, GMMs are frequently employed in biometric systems, such as speaker recognition systems. The GMM’s capacity to produce smooth approximations to arbitrarily shaped densities is one of its most impressive features. For datasets of NDs that have significant voice data, GMMs can play a promising role in classifying them. The GMM has shown promise in medical applications, such as medical imaging [208] and identifying Parkinson’s disease [209]. Some corresponding analyses about GMM are tabulated in Table 9.

#### 7.1.3. K-Nearest Neighbors (K-NN)

The K-nearest neighbors (K-NN) is an instance-based model. The performance of the inference depends on its nearest neighbor values. The model requires less training as all training data are stored in memory and used throughout the prediction phase, which is a significant advantage. The K-most-similar neighbors to the new sample are recognized using a distance function [216]. The label of the unknown sample is the average of the labels of its closest neighbors. The K-NN classifier determines the data vectors by considering the classes and examining the diseases’ components. In some cases, where an ND has multiple classes, including mild, severe, or healthy, the K-NN classifier shows the efficiency. Many researchers used K-NN and FK-NN models to diagnose neurological illnesses [217]. Fuzzy K-NN (FK-NN) is a more advanced approach that has been used to diagnose Parkinson’s disease using computational speech analysis [218,219]. There are some corresponding analyses about KNN that are tabulated in Table 10.

#### 7.1.4. Generative Adversarial Networks (GAN)

A contemporary ML approach is the generative adversarial network (GAN). The two ANN models competed in training each other simultaneously in the GAN. Machines can use GANs to imagine and develop new images independently. It has been evaluated in contexts of medical image synthesis [224] and patient-record production [225]. There are some related analyses about KNN that are included in Table 11.

#### 7.1.5. Random Forests (RF)

RF is a decision tree-based ensemble approach. Ensemble methods produce a more efficient prediction model by combining the results of various learning algorithms. Each RF tree was constructed using a random subset of the training data and the characteristics that improve the generalization and robustness to outliers. The final estimation is the average or the majority of the trees’ calculations, whether the goal is a regression or classification task [231]. There are some result analyses about RF that are included in neurological disease detection in Table 12.

#### 7.1.6. Artificial Neural Network (ANN)

A neural network (artificial neuron network) is a computational model that shows how nerve cells work in the brain. The term “ANN” refers to parallel architecture inspired by how biological neural processing works. Artificial neural networks (ANNs) use learning algorithms that can make adjustments or learn on their own as new information is received. As a result, they are an excellent tool for non-linear statistical data modeling [234,235,236]. The multi-layer feed-forward neural network is popular among various ANN architectures. The L-M algorithm is efficient and strongly recommended for neural network training for small- and medium-sized networks, according to Hagan et al. [237]. There are some result studies about ANN that are tabulated in NDs detection in Table 13.

### 7.2. Deep Neural Network Algorithms

**Figure 4:**
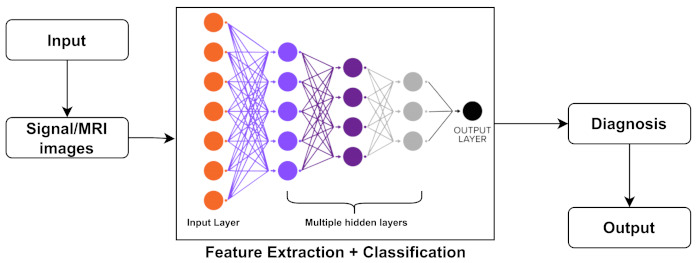
Representation of common deep neural network structure.

Deep learning (DL) is a more advanced tool for machine learning (ML) systems, which is a subset of artificial intelligence (AI) in the computer science field [242]. In other words, DL is considered a branch of machine learning that can be used to create models that extract high-dimensional characteristics from data. It has gotten a lot of attention in recent years, notably in the field of image analysis. A common structure of image analysis or the classification of DL methods is shown in Figure 4. In addition, a brief discussion on DL classifiers is represented further.

#### 7.2.1. Convolutional Neural Network (CNN)

CNN’s have performed admirably in various computer vision and pattern recognition tasks recently. CNNs sparked significant interest after it won the ImageNet [243,244] competition in 2012, although it was first published in 1989 [245]. This achievement can be attributed to extracting fundamental spatial qualities from raw data. CNNs can easily define data without requiring human intervention in feature selections [246]. The convolution, pooling, and fully linked are the three primary layers of a CNN. CNNs perform considerably better than the previous highest-computing algorithms on a dataset of approximately one million photos containing thousands of different classifications. CNNs are feed-forward neural networks usable in image processing, pattern recognition, and classification problems. The visual cortex’s biological mechanism influenced this architecture. That is why this architecture works far better in MRI image processing, symptomatic pattern detection, or the classification of NDs. The convolution layer filters the input data, such as kernels with trainable parameters, to create the feature map. The feature map is then down-sampled using the pooling layer to reduce the dimension and consequently the computational complexity and overfitting. These settings enabled the learning of many network features while keeping the number of traceable parameters low. The CNN has fewer specialized jobs than typical deep learning systems and learns to extract features thoroughly. There are some result studies about CNN that are tabulated in NDs detection in Table 14.

#### 7.2.2. Recurrent Neural Network (RNN)

Recurrent neural networks (RNNs) [253] are a type of artificial neural network that can hold a state across numerous sequential inputs. The primary purpose is to assess the temporal sequence of data points using computations from previous sequences. RNNs contain a memory that keeps track of their present state, making them perfect for forecasting time-series signals, such as EEGs. However, problems including exploding and vanishing gradients and information morphing are common when using back propagation to train RNNs. There are some result investigations regarding RNN that are tabulated in NDs in Table 15.

#### 7.2.3. Long–Short-Term Memory (LSTM)

Long–short-term memory networks (LSTMs) [256] are a variety of RNNs used to learn long-term dependencies. LSTMs tackle the problem of exploding and disappearing gradients by preserving the mistake that is back propagated through layers and time. Long–short-term memory units (LSTM) aid in disseminating knowledge times. The regulating gates of an LSTM cell may store in, erase from, write to, and read from cells. An LSTM cell creates two states with each time step: a cell state that acts as the input to the next step and a concealed condition that results from this time step. Some of the result analysis regarding RNN are tabulated in NDs in Table 16.

#### 7.2.4. Extreme Learning Machine (ELM)

Huang et al. [264] created an extreme learning machine (ELM). The ELM is a hidden layer feed-forward neural network with randomly computed input weights and analytically determined output weights. The ELM hidden layer uses sigmoidal, Gaussian, and hard-limited activation functions, whereas the output layer uses a linear process. In comparison to feed-forward networks that learn using the backpropagation method, ELM has a higher generalization success rate. References [265,266,267] describe ELM’s learning algorithm. Some of the result analysis regarding ELM are tabulated in NDs in Table 17.

#### 7.2.5. Gated Recurrent Unit (GRU)

The GRU is an LSTM variant that merges the input and forget gates into a single update gate. It combines the input and forget gates and makes some other changes. The number of gate signals was reduced to two: the reset gate and update gate signals. These two gates determine the data that must be sent to the output. Some of the result analysis regarding GRU are tabulated in NDs in Table 18.

#### 7.2.6. Deep Boltzmann Machine (DBM)

The deep Boltzmann machine (DBM) is a 2009 generative and unsupervised learning system comprising stacked layers of RBM. Similarly, the deep Boltzmann machine (DBM) was built by combining numerous RBMs. An associative memory layer converts the DBM model to the DBN model (at the top of the DBM). All layers of the DBM architecture are linked in an undirected manner. The DBM technique, similar to a DBN, can handle ambiguous inputs and sophisticated internal representations of input data in a robust way. Extensive applications of the DBM include object and speech recognition, which can work better in detecting PDs with its speech data. Some of the result analysis regarding DBM are tabulated in NDs in Table 19.

#### 7.2.7. Deep Belief Networks (DBNs)

Restricted Boltzmann machines (RBMs) are undirected graphical models representing variations of deep Boltzmann machines (DBMs). Unconstrained Boltzmann machines may be linked to concealed units. The RBMs were stacked to produce a DBN, and RBM was the building block of the DBN. The DBNs are unsupervised probabilistic hybrid generative DL models with latent and stochastic variables. Furthermore, the convolutional DBN (CDBN) is a variant of a DBN that can successfully scale a high-dimensional model utilizing adjacent pixels’ spatial information. DBNs are probabilistic, generative, unsupervised DL models with visible and hidden units in many layers. Some of the consequences of studies regarding DBNs are tabulated in NDs in Table 20.

#### 7.2.8. Probabilistic Neural Network (PNN)

The PNN is a pattern recognition and classification algorithm. First, a Parzen window with a negative function estimates the probability distribution function (PDF) in the PNN. Next, the PDF function was used to calculate the likelihood of new input data. Finally, the new input data are assigned to the class with the highest posterior probability using the Bayes rule. This strategy aids in reducing the amount of data misclassification.

#### 7.2.9. Autoencoders (AEs)

The AE model is an unsupervised machine learning model in which the input and output are the same. The input is compressed into a latent-space representation and used to generate the output. As a result, the neural network and compression and decompression functions are linked in the AE. The encoder, code, and decoder are the three components of an AE. AE networks are most often employed in brain signal processing for feature extraction or dimensionality reduction.

These classification algorithms are ordered by the most commonly utilized for detecting NDs. Between these machine learning algorithms, the SVM, KNN, and Naive Bayes are the most widely used methods and researchers’ first choices, as they give a better memorization performance in the first phase of utilizing the models, thereby assuring the performance of the models. However, These algorithms are not always suitable, as some are not good enough for more extensive datasets. Therefore, there is some scope for developing classification algorithms for real-time brain signal analysis. In deep learning algorithms, the CNN, LSTM, GRU, and DBM have proven to be better in the study and detection of neurological diseases. However, researchers improve deep learning algorithms as they show better performance on time series and brain image data. Furthermore, transfer learning and attention models were recently utilized in different studies to bring better performance in extensive dataset analyses. In this section, we tried to establish a relation between the most advantageous techniques of pre-processing, feature extraction, and classification, representing a CAD system that detects or diagnoses NDs. The quality measures of the system are illuminated in the following section.

## 8. Evaluation Metrics

Evaluating a model is essential for developing a practical machine learning and deep learning model. After pre-processing, training, and validation, the test images were sent to the trained model for classification to evaluate its performance. The confusion matrix, cross-validation, receiver operating characteristic curve (ROC), the area under the ROC curve (AUC), and other evaluation metrics exist. The confusion matrix’s accuracy, precision, recall, and F1-score are commonly used to evaluate the model of ND classification.

### 8.1. Accuracy

The accuracy metric measured the percentage of correctly identified samples. The accuracy of the binary classification was calculated as follows:

(5) $$\;\mathrm{Accuracy}\;=\left(\frac{TP+TN}{(TP+TN+FP+FN}\right)$$

where true positives (*TP*) denote correct positive example assignments, true negatives (*TN*) denote correct negative example assignments, false positives (*FP*) denote incorrect positive example assignments to negative classes, and false negatives (*FN*) denote incorrect negative example assignments to positive classes.

### 8.2. Sensitivity or True Positive Rate or Recall

Sensitivity, also known as the recall and true positive rate, locates all positive samples where denotesthe activity of the classifier. Sensitivity shows the ratio of correctly classified patients with NDs to the total number of patients with NDs. The sensitivity formula is determined as follows:

(6) $$\;\mathrm{Sensitivity}\;=\frac{TP}{TP+FN}$$

### 8.3. Specificity or True Negative Rate

The true negative rate (TNR), often known as the specificity of a test, is the percentage of samples that test negative with the test in the truly negative issue. A test that identifies all healthy persons as negative for a given condition, for example, is exceedingly specific. In other words, the percentage of accurately diagnosed healthy persons across the entire range of healthy people is referred to as specificity.

(7) $$\;\mathrm{Specificity}\;=\frac{TN}{TN+FP}$$

### 8.4. Precision

The precision metric calculates the percentage of relevant issues that are relevant. It assesses a classifier’s ability to reject irrelevant subjects. The recall metric measures the number of relevant subjects that are discovered. It considers the classifier’s ability to present all relevant subjects. The figures are written as follows:

(8) $$PR=\left(\frac{TP}{TP+FP}\right)$$

### 8.5. F1-Score

The *F*1-Score computes the harmonic mean between the precision and recall by grouping together with their values. The formula for *F*1-score is defined as:

(9) $$F1-Score=\left(\frac{2*Precision*Recall}{Precision+Recall}\right)$$

### 8.6. Mcc

MCC represents a classifier’s ability to anticipate and generate a value between [1, +1]. If the MCC of the classifier is +1, then the classifier produces perfect predictions. Classifiers with a value of one have utterly incorrect outputs. Closer MCC values imply that the classifier makes random predictions.

(10) $$\mathrm{MCC}=\frac{TP\times TN-FP\times FN}{\sqrt{\left(TP+FP)(TP+FN)(TN+FP)(TN+FN\right)}}$$

### 8.7. Roc Curve and AUC

The receiver operating characteristics (ROC) curve is used to demonstrate the effectiveness of the classification model over several categorization levels. In this curve, the true-positive rates (recall) and the false-positive rates (*FPR*) were employed. The term “area under the ROC curve” is abbreviated as “AUC”. In other words, the AUC evaluates the entire two-dimensional field under the ROC curve. The formula is used as follows:

(11) $$FPR=\frac{\;\mathrm{FalsePositive}\;}{\;\mathrm{FalsePositive}\;+\;\mathrm{TrueNegative}\;}$$

### 8.8. Patient Score

If $N_{\mathrm{rec}}$ cancer photos are successfully recognized for each patient and $N_{P}$ is the total number of cancer images for patient *P*, a patient score can be defined as in Equation (12) as follows:

(12) $$\;\mathrm{PatientScore}\;=\frac{N_{\mathrm{rec}\;}}{N_{P}}$$

### 8.9. Patient-Recognition Rate

The patient-recognition rate is defined by the formula as follows:

(13) $$\;\mathrm{PatientRecognitionRate}\;=\frac{\sum \;\mathrm{PatientScore}\;}{\;\mathrm{Total}\;\mathrm{Numberof}\;\mathrm{Patients}\;}$$

### 8.10. Image-Recognition Rate

The number of cancer photos in the test set was represented by $N_{\mathrm{all}}$. The recognition rate at the image level may be represented by Equation (14) if the system correctly classifies $N_{\mathrm{rec}}$ cancer photos.

(14) $$\;\mathrm{Image}\;-\;\mathrm{recognitionrate}\;=\frac{N_{\mathrm{rec}\;}}{N_{\mathrm{all}\;}}$$

These evaluation metrics are the primarily utilized techniques and are arranged in importance for detecting NDs by researchers. With this section, the research led to the challenges faced while researching neuron-related disorders.

## 9. Challenges and Opportunities

As previously noted, AI has played a crucial role in identifying neurological illnesses. It has transformed the massive amounts of data collected into clinically relevant data [286]. However, there were significant restrictions and uncertain legal repercussions despite these advantages. Even with the most powerful algorithms, it is impossible to account for all the potentially beneficial or adverse side effects [287]. DL algorithms prefer to minimize adverse reactions and confusing factors, such as test results, to reach their aim. This can impair patient safety and outcomes [287]. Table 21 presents several advantages and the limitations of the DL and ML methods. Additionally, different reasoning methods, or a combination of approaches, such as case-based, rule-based, model-based, fuzzy logic, genetic algorithms, natural language processing, and neural networks, have been used by various computers in a literature review. Each method has its capabilities and limitations [288]. The efficacy of each technique differed, limiting their application in detecting rare and complex disorders, such as multiple sclerosis [288]. Nonetheless, they could assist patients and clinicians in making a rapid clinical diagnosis [288].

### 9.1. Mostly Faced Challenges

DL-based frameworks for NLD prediction have become attractive with the significant growth in computational capacity and the remarkable development of DL tools. However, more studies should be performed to fine-tune DL algorithms to improve inferences. The following are some of the concerns, along with potential prospects.

#### 9.1.1. Lack of Standard Data

In machine learning, data standards and open data repositories are lacking. For instance, the non-integration of motor and non-motor features and the lack of open data storage and available programming have hampered the integration of existing commercial medical instruments, such as Parkinson’s Kinetigraph TM, which have moderate healthcare coverage [289].

#### 9.1.2. Small Sample Size

The ML view implies that sample sizes ought to be a multiple of the number of input and output variables [290]. However, studies have been conducted using small sample sizes of patients [291,292,293]. Creating an extensive dataset of patients with these mental disorders can be highly beneficial to physicians for accurately diagnosing diseases. Further attention and more practical research in this field can fulfill the necessity for longitudinal studies or a follow-up study on a patient’s transition.

#### 9.1.3. DL Algorithms Need a Large Trained Dataset

For massive datasets, DL algorithms provide impactful and accurate solutions. However, high-dimensional CNNs such as 2D-CNN and 3D-CNN for big and multimodal neuroimages will yield high accuracy. A generative adversarial network (GAN) can create a synthetic neuroimage utilized with a CNN. The availability of training datasets was one of the most significant impediments to the application procedure of DL in neuroimaging, which also comes in as a consequence of maintaining patient privacy. Simultaneously, annotating these data is a significant challenge that necessitates expert assistance. As a result, the dataset for uncommon diseases discovered was mainly unbalanced. Medical practitioners and data analysts must collaborate to solve dataset development and annotation challenges. Simultaneously, data augmentation techniques can be used to solve the problem of unbalanced data by altering the volume and quality of the data

#### 9.1.4. Bias-Free Neuroimaging Dataset

It is challenging to construct a bias-free neuroimaging dataset because it is a legacy of a learning system that could result in a computational artifact. However, the risk can be addressed by incorporating an extensive dataset into the model, examining the link between the extracted features and fine-tuning the model’s parameters.

#### 9.1.5. Limitation of ML Clinical Presentation

The clinical significance of ML in confounding groups with similar neurological, psychological, or pathological manifestations are limited: for example, ML’s ability to differentiate PNES not only from epileptic patients but also from patients based on psychopathological displays, including major depression, or ML’s ability to discern epilepsy from healthy controls versus application in patients with a panic disorder [294], schizophrenia [295], and drug-induced memory deficits with similar alterations in microstate C.

#### 9.1.6. Non-Standardized Acquisition of Images

This resulted in discrepancies in the photos from different databases. It is a significant challenge when using DL to evaluate neuroimages. To solve this difficulty, it is suggested that transfer learning be used. These extensive training datasets are considered the foundation for attaining better results with ML and DL techniques. The lack of these datasets is one of the most significant impediments to the application process.

#### 9.1.7. DL Models Are Black Box

A deep learning model is a black box that learns from data and models the data collection process. Instead of being explainable, these models can be interpreted. However, when the model is used to forecast with data that do not belong to the database, the black box fails miserably. When the mechanism is employed to foresee an explainable process, according to Rudin, DL is overly sophisticated, highly recursive, and challenging to comprehend [296]. Consequently, the explanation frequently fails to provide sufficient information to understand the DL mechanism. As a result, there is a frequent transition between explainable and interpretable DL models.

#### 9.1.8. Ethical and Legal Ramifications

While the ethical and legal implications are beyond the focus of this work, maintaining patient trust would be critical in supporting collaboration and AI application [297]. AI-enabled computer-aided diagnostic (CAD) solutions [298] are unfeasible in black-box situations. The legal implications are unclear, especially whether the manufacturer or the practitioner is to blame [299]. Therefore, standards for evaluating AI technologies must be developed. It is debatable whether AI can replace doctors. Nonetheless, AI will play a more significant role in integrating healthcare.

#### 9.1.9. Limitation of Supervised Architecture

Considering the time and labor necessary to manufacture label data, low scalability, and the selection of optimal bias levels, the supervised architecture was excluded. For image analyses, unsupervised learning was not a standard option. On the other hand, unsupervised architecture learns features from a dataset. It creates a data-driven decision support system from it. Consequently, an unsupervised deep architecture can overcome medical imaging-related issues.

#### 9.1.10. Adversarial Noise

It can enhance neuroimages while also lowering classification accuracy. As a result, canceling adversarial errors is difficult.

#### 9.1.11. Lack of Sufficient Hardware Resources

The most significant aspect of CAD is the identification of differentiating traits that can lead to the identification of the valuable bio-markers of NDs. In addition, owing to a lack of access to hardware resources and data availability, developing DL architectures to diagnose NDs is challenging. Although tools such as Google Colab and others now provide researchers with powerful computing processors, implementing and using these methodologies in the real world still presents numerous challenges [300].

### 9.2. Future Research Directions

To overcome the existing challenges, approaches based on graph theory and machine learning have quickly emerged as a significant trend to understand better paths to appropriately diagnose and adequately handle disorders given the availability of high-dimensional data and increased computing capacities. Under the umbrella of machine learning-based solutions to neurological illnesses, deep learning has recently gained an ever-increasing position in the era of health and medical studies. Our best hope for treating neurological diseases in children is to combine applied deep learning with graph theory on a tailored scale. However, doctors or clinicians must practically diagnose mental disorders by acknowledging symptoms. These symptoms can be very similar to other diseases, which can be confusing. For example, symptoms such as difficulties in movements and memory or awareness loss were identical in several NDs. This is why doctors must be particular in distinguishing diseases. DL in neuroimaging is also derived from the desire to protect patient privacy. Simultaneously, annotating these data is a significant issue that necessitates expert assistance. As a result, the datasets discovered for uncommon diseases are typically unbalanced. These data primarily depend on brain signals that contain noise and artifacts. Therefore, the health industry, medical practitioners, and data scientists must solve dataset development and annotation challenges. Simultaneously, data augmentation techniques can be used to address the issue of unbalanced data by altering data volume and quality [10].

Some other aspects need to be handled in the case of the neurological disorder detection directions. These are indicated in the following section.

#### 9.2.1. Deep Brain Stimulation (DBS)

It is a safe and effective surgical therapy option for patients with tremor manifestations, including Parkinson’s disease (PD) [301,302] and essential tremor (ET) [303,304]. A neurologist currently determines DBS leads, and as a result, interpersonal variability is a factor. AI could assist in making an objective analysis in this aspect, provided that regulatory standards are met and medical clearance is obtained [291].

#### 9.2.2. Open Data Portals

Open data portals may be in people’s best interests [86]. Study models, frameworks, algorithms, and anonymous data samples have all been made open source by some researchers, making it easier to replicate in future investigations [291]. The use of low-cost smartphones with widely available customized apps boosts their usefulness [291].

#### 9.2.3. Testing Multiple Hypotheses

Compared with human skills, newer algorithms can test several hypotheses in an acceptable amount of time and make prior assumptions [287]. It can be used to treat various diseases and specialties [287]. ML can evaluate massive datasets at considerably faster rates with an improved accuracy. ML methods such as the self-organizing map (SOM) can be extended to include non-vestibular factors, including previous concussions, neuropsychological results, and various other variables [305]. It also aids in the improvement of diagnostic criteria by identifying characteristics across a wide range of patient populations [306].

#### 9.2.4. Utilizing Methodology in Brain Signal Analysis

Researchers still could not figure out the ultimate solution for brain signal analysis. There is the requirement for making brain signals readable by using a method that can lead us to the solution, which is identifying the early stage diseases that can occur in the brain or neuron. There is massive scope for improving the classification methods in terms of the neurological disease detection process.

We must establish the best practices for evaluating AI tools [307]. It is uncertain whether AI will replace physicians, but it will undoubtedly play a more prominent role in health care integration [194].

## 10. Conclusions

Advancements in high-speed computing techniques and remarkable improvements in creating novel DL-based methods and models open up unique possibilities for predicting and maintaining several neurological disorders. A comprehensive assessment of NDs and their symptoms are exhibited in this study. The study also includes several works on NDs, the datasets utilized in most detection procedures, and neurological modalities. This paper summarizes the present methods for creating various types of NDs systems. Some popular pre-processing techniques with machine learning and deep learning models were reviewed thoroughly. Moreover, we can apply many neurological modalities to detect early-stage neurological diseases. In that circumstance, the patient might begin taking medication right away to fight the disease. More future studies are needed in this sector that integrate various screening approaches to diagnose neurological diseases precisely and quickly. Researchers should focus on embedded applications that enhance the accurate identification of such disorders.

## Figures and Tables

**Table 1:** Neurological diseases-related recent surveys summary.

Ref.	Purposes	Challenges
[3]	This study discovered various deep learning algorithms for diagnosing epilepsy, stroke, PD, and AD on EEG- and MRI-based data	The application of deep learning techniques in diagnosing additional neuropsychiatric and neurological illnesses, aside from those stated, was not considered during the meta-analysis synthesis.
[7]	This study reflects on segmentation, classification, and prediction of brain tumors using deep learning techniques	Some challenges include labeling images of tumors and label uncertainty directly in the loss function.
[8]	This study addressed the performance and deficiencies of deep learning-based brain tumor classification (BTC) with various pre-processing, feature extraction, and classification techniques	Lack of large training dataset; class imbalance due to data augmentation.
[9]	This study investigated automated epileptic seizure identification using DL approaches and modalities, such as neuroimaging, EEG, and MRI.	Inaccessibility of datasets with long registration times, and the datasets used to diagnose epileptic seizures have a finite registration period; conducting essential research on the subject of epileptic seizures.
[10]	This study showed an overview of different DL and pre-processing strategies for detecting anomalies of, and the diagnosis and classification of AD, PD, and SZ with various open-access MRI data.	Predicting NLD in real-time from imaging data; developing a bias-free neuroimaging dataset; and adding adversarial noise to the neuroimages can reduce classification accuracy.
[12]	This study utilizes raw embryo brain images to develop three deep convolutional neural networks (DCNNs) with distinct architectures	Not focused on common neurological diseases.

**Table 2:** A tabulation of popular datasets of Alzheimer’s disease.

Database Name	Healthy Control(HC)/Patient(P)	Modality	Available in (Last Access Date)
Alzheimer’s Disease Neuroimaging Initiative (ADNI)	P: 47 HC: 34	MRI	http://adni.loni.usc.edu/about/ (5 January 2022)
Open Access Series of Imaging Studies (OASIS) 1	S: 416	MRI	https://www.oasis-brains.org/ (5 January 2022)
OASIS 2	S: 150	MRI	https://www.oasis-brains.org/ (5 January 2022)
OASIS 3	S: 1098	MRI & PET	https://www.oasis-brains.org/ (5 January 2022)
Chosun University Hospital (GUH) and Gwangju Optimal Dementia left (GODC) [13]	HC: 10 P: 10	EEG	NA

**Table 3:** A tabulation of popular datasets of Parkinson’s disease.

Database Name	Healthy Controls(HSC)/ Patient(P)	Modality	Available in (Last Access Date)
Sprial Dataset (UC Irvine Machine Learning Repository)	P: 62 HC: 15	Handwriting	https://archive.ics.uci.edu/ml/datasets/Parkinson+Disease+Spiral+Drawings+Using+Digitized+Graphics+Tablet (7 January 2022)
Shanghai East Hospital of Tongji University (TCS Dataset)	P: 76 HC: 77	Ultrasound Images	https://www.aimspress.com/article/doi/10.3934/mbe.2019280?viewType=HTML (7 January 2022)
Dandenong Neurology Centre, Melbourne, Australia [14]	HC: 40 P: 41	NA	NA
Parkinson’s Progression Markers Initiative (PPMI)	P: 498 HC: 203	Images	https://www.ppmi-info.org/access-data-specimens/data (7 January 2022)
Parkinson’s Disease Classification Dataset	P: 188	Speech recordings	https://archive.ics.uci.edu/ml/datasets/Parkinson%27s+Disease+Classification (7 January 2022)
Parkinsons Dataset	P: 23	Voice recording	https://archive.ics.uci.edu/ml/datasets/parkinsons (7 January 2022)
Parkinsons Telemonitoring Voice Dataset	P: 42	Speech Recordings	https://archive.ics.uci.edu/ml/datasets/parkinsons+telemonitoring (7 January 2022)

**Table 4:** A tabulation of popular datasets of Cerebral palsy.

Database Name	Healthy Control(HC)/ Patient(P)	Modality	Available in (Last Access Date)
Dataset cerebral Palsy Pre- and Post-Botulinum Toxin A [15]	P: 49	NA	https://figshare.com/articles/dataset/Dataset_cerebral_palsy_pre_and_post_Botulinum_Toxin_A2055729 (8 January 2022)

**Table 5:** A tabulation of popular datasets of brain tumor.

Database Name	Healthy Control(HC)/ Patient(P)/Images(I)	Modality	Available in (Last Access Date)
Brain MRI Images for Brain Tumor Detection	I: 253	MRI	https://www.kaggle.com/navoneel/brain-mri-images-for-brain-tumor-detection (10 January 2022)
Sample Brain Tumor Dataset	NA	MRI	https://ieee-dataport.org/documents/brain-tumor-dataset (10 January 2022)
Brain Tumor Dataset	I: 3064	MRI	https://figshare.com/articles/dataset/brain_tumor_dataset/1512427 (9 January 2022)
Br35H: Brain Tumor Detection 2020	I: 3864	MRI	https://www.kaggle.com/ahmedhamada0/brain-tumor-detection (9 January 2022)
BraTS 2013	P: 55	MRI	https://paperswithcode.com/dataset/brats-2013-1 (10 January 2022)
BraTS 2014	NA	MRI	https://paperswithcode.com/dataset/brats-2014-1 (10 January 2022)
BraTS 2015	I: 274	MRI	https://paperswithcode.com/dataset/brats-2015-1 (10 January 2022)
BraTS 2017	I: 285	MRI	https://paperswithcode.com/dataset/brats-2017-1 (10 January 2022)
BraTS 2018		NA	MRI https://paperswithcode.com/dataset/brats-2018-1 (10 January 2022)
BraTS 2019	NA	MRI	https://paperswithcode.com/dataset/brats-2019-1 (10 January 2022)

**Table 6:** A tabulation of popular datasets of epilepsy.

Database Name	Healthy Control(HC)/ Patient(P)/Sample(S)	Modality	Available in (Last Access Date)
Bonn Time Series Satabase	NA	EEG	https://repositori.upf.edu/handle/10230/42894 (12 January 2022)
Bern–Barcelona	S: 10,240	EEG	https://www.upf.edu/web/ntsa/downloads (12 January 2022)
Temple University EEG corpus	P: 10,874	EEG	https://isip.piconepress.com/projects/tuh_eeg/html/downloads.shtml (12 January 2022)
Neurology and Sleep left, New Delhi EEG Database	S: 1024	EEG	https://www.researchgate.net/publication/308719109_EEG_Epilepsy_Datasets (12 January 2022)
Children Hospital Boston, Massachusetts Institute of Technology (CHB-MIT) [16]	P: 22	EEG	https://physionet.org/content/chbmit/1.0.0/ (12 January 2022)
Siena Scalp [17]	P: 14	EEG	https://physionet.org/content/siena-scalp-eeg/1.0.0/ (12 January 2022)
Single Electrode Data	HC: 15 P: 15	EEG	https://zenodo.org/record/3684992#.YYg27GBBzDd (12 January 2022)
Epileptic Dataset	P: 6	EEG	https://data.mendeley.com/datasets/5pc2j46cbc/1 (12 January 2022)
A Dataset of Seizures Annotations	NA	EEG	https://zenodo.org/record/1280684#.YYg3v2BBzDd (12 January 2022)

**Table 7:** This is a dataset for symptoms and detection modalities for different neurological diseases.

Disease Name	Symptoms	Detection
Parkinson’s disease	Tremor, sluggishness of movement, stiff muscles, uneven gait, and balance and coordination issues are symptoms of Parkinson’s disease.	Movement, speech, neuroimaging, handwriting patterns, cerebrospinal fluid (CSF), optical coherence tomography (OCT), magnetic resonance imaging (MRI), and single-photon emission computed tomography (SPECT).
Dementia	Memory loss, difficulty with tasks, confusion, language issues, behavioral changes, and a loss of initiative are all symptoms of Alzheimer’s disease.	Listening to medical history, evaluating cognitive performance and mental state, neuropsychological testing, assessing daily activities, clinical laboratory tests, and brain imaging testing.
Alzheimer’s disease	Early stage: Memory lapses, such as forgetting standard terms or where everyday objects are. Middle stage: Misunderstand statements, become upset or furious, and act strange, such as refusing to bathe. Damage to nerve cells makes it difficult for people to express their thoughts and do ordinary tasks without help. Late stage: Lose awareness of their surroundings as well as recent experiences. Get into trouble walking, sitting, swallowing, and communicating, etc. Become more susceptible to infections, including pneumonia.	Raw neuroimaging modalities for combinatorial measures, such as sub-cortical volumes, gray matter densities, cortical thickness, brain glucose metabolism, and cerebral amyloid.
Multiple sclerosis	Fatigue, difficulty walking, stiffness, weakness, vision issues, dizziness, cognitive changes, emotional changes, and sadness, etc., can occur.	MRI scan, as radio waves and magnetic fields are used in it to assess the relative water content of bodily tissues to distinguish between normal and pathological tissues.
Cerebral Palsy	Delays in development, irregular muscular tone, and poor posture are all common.	X-ray computed tomography (CT scan) and magnetic resonance imaging (MRI) are two brain imaging procedures. An electroencephalogram (EEG), genetic testing, and metabolic testing are also performed.
Brain Tumor	Some symptoms include headaches, seizures, visual and speech issues, memory loss, and loss of balance.	A brain tumor is usually diagnosed in three steps: An examination of the nervous system. Brain scans include CT (or CAT) scans, MRIs, angiograms, X-rays, and others. A biopsy is a procedure that is used to examine (tissue sample analysis).
Epileptic seizures	Uncontrollable jerking motions of the arms and legs, temporary disorientation, stiff muscles, consciousness or awareness loss, and fear and anxiety.	EEG, EMG, ECG, motion, or audio/video recording on the human head and body are used to monitor brain and muscle activities, heart rate, oxygen level, artificial sounds, or visual signatures.

**Table 8:** A tabulation of result analysis of SVM classifier.

Ref.	Dataset	Evaluation Metrics	Methods	Accuracy
[199]	EEG Dataset [200]	Epilepsy	Accuracy, Sensitivity, Specificity	Above 95%
[201]	CP and Normal Children’s Gait Data [202]	Cerebral Palsy	Accuracy, Sensitivity, Specificity	Above 82%
[203]	3D Brain MR Image	Multiple Sclerosis	Accuracy, Sensitivity, Specificity	0.996
[204]	ADNI	Alzheimer Disease	Precision, Recall, F-measure	96.63%
[205]	Brain Tumor MR Images from Kaggle	Brain Tumor	Accuracy	92%
[206]	U/I	Dementia	Accuracy, Sensitivity, Specificity, F1-Score, Precision, MCC	92.36%
[207]	Parkinson’s Disease Handwriting Data (NewHandPD)	Parkinson’s Disease	Accuracy, Sensitivity, Specificity, F1-Score	77.45%

**Table 9:** GMM classifier result analysis on various NDs.

Ref.	Dataset	Evaluation Metrics	Methods	Accuracy
[210]	Cancer-Imaging Archive	Brain Tumor	Accuracy, AUC	94.11%
[211]	Image from Longitudinal MS Lesion Segmentation Challenge	Multiple Sclerosis	Dice Similarity Coefficient (DSC), True Positive Rate (TPR), False Positive Rate (FPR), Volume Difference (VD) and Pearson’s r Coefficient	DSC: 0.62
[212]	Epileptic EEG dataset [200]	Epilepsy	Accuracy	99%
[213]	Dataset from Department of Neurology in Cerrahpaşa Faculty of Medicine, Istanbul University [214]	Parkinson’s Disease	Accuracy, MCC	89.12%
[215]	SPECT Datasets from Clinic of Nuclear Medicine, University of Erlangen-Nuremberg	Dementia	Accuracy	93.39%

**Table 10:** KNN classifier result analysis on various NDs.

Ref.	Dataset	Disease	Evaluation Metrics	Accuracy
[220]	Collected from Clinical Courses	Multiple Sclerosis	F1-Score, Precision, Accuracy	F1: 81%
[217]	PD Dataset [221]	Parkinson’s Disease	Sensitivity, Specificity, Accuracy	96.07%
[222]	EEG Dataset	Epilepsy	Sensitivity, Specificity, Accuracy	Above 95%
[223]	Hyperspectral Brain Cancer Image Database	Brain Tumor	Euclidean & Manhattan distance	U/I

**Table 11:** GAN classifier result analysis on various NDs.

Ref.	Dataset	Disease	Evaluation Metrics	Accuracy
[226]	ADNI Dataset	Alzheimer’s Disease	Accuracy, Recall, Precision, F-2	94.1%
[227]	ADNI & NIFD	Alzheimer’s Disease	Accuracy, Sensitivity	87.80%
[228]	CHB-MIT, Freiburg Hospital & EPILEPSIAE Dataset	Epileptic Seizure	AUC	above 80%
[229]	UCI Dataset [230]	Parkinson’s Disease	Accuracy, Sentivity, Specificity	91.25%

**Table 12:** RF classifier result analysis on various NDs.

Ref.	Dataset	Disease	Evaluation Metrics	Accuracy
[232]	Parkinson’s Disease Dataset [221]	Parkinson’s Disease	Accuracy, Kappa, Precision, Recall, AUC, F-measure	94.89%
[233]	Collected from Cerebrum Web Informational Index	Brain Tumor	Sensitivity, Specificity, Accuracy	98.37%

**Table 13:** ANN classifier result analysis on various NDs.

Ref.	Dataset	Disease	Evaluation Metrics	Accuracy
[238]	Clinical & HRV data	Cerebral Palsy	AUC	>90%
[239]	Independent Samples	Multiple Sclerosis	ROC curve	94.5%
[240]	Population-Based Nested Case-Control Study Sesign	Alzheimer’s Disease	Sensitivity, Specificity, Accuracy, AUC	92.13%
[241]	University of California at Irvine (UCI) Machine Learning Repository	Parkinson’s Disease	Accuracy, Sensitivity, Specificity, MCC	86.47%

**Table 14:** CNN classifier result analysis on various NDs.

Ref.	Dataset	Disease	Evaluation Metrics	Accuracy
[247]	MR Image Dataset [248]	Seizure Detection	Sensitivity, Specificity, Accuracy, Precision, F-Score	96.05%
[249]	MRI Dataset from McGill University	Cerebral Palsy	Accuracy	88.6%
[250]	Parkinson’s Disease Spiral Drawings Using Digitized Graphics Tablet Dataset [251]	Parkinson’s Disease	Accuracy, AUC, F1-Score	96.5%
[252]	OASIS	Alzheimer’s Disease	Accuracy	78.02%

**Table 15:** RNN classifier result analysis on various NDs.

Ref.	Dataset	Evaluation Metrics	Methods	Accuracy
[254]	Daphnet Dataset	Parkinson’s Disease	AUC, Specificity, Sensitivity	Avg 97%
[255]	ADNI	Alzheimer’s Disease	Accuracy, Specificity, Sensitivity	89.69%

**Table 16:** LSTM classifier result analysis on various NDs.

Ref.	Dataset	Evaluation Metrics	Methods	Accuracy
[257]	VGRF Dataset [258]	Parkinson’s Disease	Accuracy, Specificity, Sensitivity, MCC, PVV, F-Score	98.60%
[259]	Molecular Brain Neoplasia Data (REMBRANDT) [260,261]	Brain Tumor	Accuracy	86.98%
[262]	ADNI	Alzheimer’s Disease	Accuracy	Above 85%
[263]	MINI-RGBD, RVI-25	Accuracy, Specifity, Sensitivity, PR, F1-Score	Above 91%	

**Table 17:** ELM classifier result analysis on various NDs.

Ref.	Dataset	Disease	Evaluation Metrics	Accuracy
[268]	EEG Dataset [269]	Epilepsy	Accuracy, Sensitivity, Specificity	90%
[270]	ADNI Dataset	Alzheimer’s Disease	Accuracy, Sensitivity, Specificity	76.9%
[271]	Epileptic EEG Dataset [200]	Epilepsy	F-Measure, G-Means, AUC	82.77%
[272]	Brain Tumor MRI Image Dataset [273]	Brain Tumor	Accuracy	94.23%
[274]	Parkinson’s Dataset [221]	Parkinson’s Disease	Accuracy, Sensitivity, Specificity, (AUC)	96.47%
[275]	Collected Data from the Vasei Hospital in Sabzevar	Multiple Sclerosis	Accuracy, Sensitivity, Specificity	97%

**Table 18:** GRU classifier result analysis on various NDs.

Ref.	Dataset	Evaluation Metrics	Methods	Accuracy
[276]	ADNI Dataset	Alzheimer’s Disease	Accuracy, Sensitivity, Specificity	97.03%
[277]	TUH EEG Seizure Corpus (TUSZ)	Epilepsy	Sensitivity, Specificity	96.9%
[278]	ADNI	Alzheimer’s Disease	Accuracy	0.709%

**Table 19:** DBM classifier result analysis on various NDs.

Ref.	Dataset	Disease	Evaluation Metrics	Accuracy
[279]	EEG Database	Seizure Detection	F-Measure, Accuracy, TPR	99.5%
[280]	Hand-Drawn Images Dataset	Parkinson’s Disease	F1-Score, Accuracy	65.62%

**Table 20:** DBN classifier result analysis on various NDs.

Ref.	Dataset	Disease	Evaluation Metrics	Accuracy
[281]	BraTS 2015	Brain Tumors	Accuracy, Recall, Precision 91.6%	
[282]	EEG Wave Files	Epilepsy Detection	Accuracy	>90%
[283]	18F-fluorodeoxyglucose-PET images	Alzheimer Disease	Accuracy, Sensitivity, Specificity	86.6%
[284]	PD Telemonitoring Dataset [285]	Parkinson’s Disease	Accuracy	94%

**Table 21:** Advantages and disadvantages of classification algorithms.

Classifier Name	Advantages	Disadvantages
SVM	SVM is memory efficient and works well in high-dimensional domains and situations there is clear separation between classes.	The SVM technique is unsuitable for big datasets and does not perform well when the dataset has a high level of noise.
GMM	It does not need the presence of a subpopulation of data points. It enables the model to automatically learn the subpopulations.	There are numerous parameters to fit, and getting decent results generally necessitates a lot of data and several iterations.
KNN	KNN is simple to install and fast because it stores the training dataset and only learns from it when making real-time predictions.	It struggles with huge datasets, high dimensions, and data that is noisy.
GAN	It provides the sharpest images because of their adversarial training, and can be trained using solely backpropagation.	It is hard to train as it is non-convergence diminished gradient.
Random Forest	It can solve classification and regression issues, is relatively steady, and is less susceptible to noise.	It has complexity and long training period.
ANN	It has the ability to store information throughout the whole network and function with partial knowledge while remaining fault tolerant.	It is hardware dependent and has an inexplicable network behavior.
CNN	It is quite accurate in picture identification and recognizes the crucial aspects automatically without human intervention.	It requires a vast amount of training data and does not encode the location or orientation of the object.
RNN	An RNN remembers all information throughout time and is useful for a time series prediction.	Training an RNN is a tough undertaking that includes gradient vanishing and explosion issues.
LSTM	It offers a wide range of parameters, such as learning rates, input and output biases, and so on.	It takes longer to train, requires more memory, and is prone to overfitting.
GRU	It requires less computational power.	It has slow convergence rate and low learning efficiency.
DBN	DBN has the ability to learn features, which is accomplished by layer-by-layer learning techniques.	It does not take into account the two-dimen sional structure of the supplied image.
PNN	It generates reliable predicted target probability scores while being somewhat insensitive to outliers.	The model requires extra memory space to be stored.
DBM	It is both expressive and computationally efficient, allowing it to encode any distribution.	Takes a lot of time to calculate probabilities and adjust weight.

## Data Availability

There is no statement regarding the data.
